# Metformin's antitumour and anti‐angiogenic activities are mediated by skewing macrophage polarization

**DOI:** 10.1111/jcmm.13655

**Published:** 2018-05-04

**Authors:** Ji‐Chang Wang, Xin Sun, Qiang Ma, Gui‐Feng Fu, Long‐Long Cong, Hong Zhang, De‐Fu Fan, Jun Feng, Shao‐Ying Lu, Jian‐Lin Liu, Guang‐Yue Li, Pei‐Jun Liu

**Affiliations:** ^1^ Department of Vascular Surgery First Affiliated Hospital of Xi'an Jiaotong University Xi'an Shaanxi Province China; ^2^ Center for Translational Medicine First Affiliated Hospital of Xi'an Jiaotong University Xi'an Shaanxi Province China; ^3^ Department of Thoracic Surgery and Oncology First Affiliated Hospital of Xi'an Jiaotong University Xi'an Shaanxi Province China; ^4^ Department of Peripheral Vascular Diseases First Affiliated Hospital of Xi'an Jiaotong University Xi'an Shaanxi Province China; ^5^ Medical Imaging Department First Affiliated Hospital of Xi'an Jiaotong University Xi'an Shaanxi Province China; ^6^ Department of Neurology First Hospital of Yulin City Yulin City Shaanxi Province China; ^7^ Department of Neurosurgery People's Hospital of Qu Wo Country Linfen City Shanxi Province China; ^8^ Department of Science and Technology First Affiliated Hospital of Xi'an Jiaotong University Xi'an Shaanxi Province China; ^9^ Key Laboratory for Tumor Precision Medicine of Shaanxi Province First Affiliated Hospital of Xi'an Jiaotong University Xi'an Shaanxi Province China

**Keywords:** anti‐angiogenesis, antitumour, macrophage polarization, metformin, tumour‐associated macrophages

## Abstract

Beneficial effects of metformin on cancer risk and mortality have been proved by epidemiological and clinical studies, thus attracting research interest in elucidating the underlying mechanisms. Recently, tumour‐associated macrophages (TAMs) appeared to be implicated in metformin‐induced antitumour activities. However, how metformin inhibits TAMs‐induced tumour progression remains ill‐defined. Here, we report that metformin‐induced antitumour and anti‐angiogenic activities were not or only partially contributed by its direct inhibition of functions of tumour and endothelial cells. By skewing TAM polarization from M2‐ to M1‐like phenotype, metformin inhibited both tumour growth and angiogenesis. Depletion of TAMs by clodronate liposomes eliminated M2‐TAMs‐induced angiogenic promotion, while also abrogating M1‐TAMs‐mediated anti‐angiogenesis, thus promoting angiogenesis in tumours from metformin treatment mice. Further in vitro experiments using TAMs‐conditioned medium and a coculture system were performed, which demonstrated an inhibitory effect of metformin on endothelial sprouting and tumour cell proliferation promoted by M2‐polarized RAW264.7 macrophages. Based on these results, metformin‐induced inhibition of tumour growth and angiogenesis is greatly contributed by skewing of TAMs polarization in microenvironment, thus offering therapeutic opportunities for metformin in cancer treatment.

## 1 INTRODUCTION

Compelling evidences that the antidiabetic metformin reduces cancer risk and cancer‐related mortality have increasingly attracted the attention of researchers.^1^, ^2^, ^3^ In the last decade, researchers are performing clinical trials to evaluate the benefits of metformin for numerous early‐stage and late‐stage malignant tumours (https://clinicaltrials.gov/). Recently, a large number of pre‐clinical studies have been or are being carried out to elucidate the mechanisms underlying metformin's beneficial effects.^4^, ^5^, ^6^, ^7^

As is reported, metformin exerted significant direct effects on inhibition of tumour cell functions in vitro, usually at concentrations equal or higher than 2 mmol/L.^8^, ^9^ Besides, high concentrations of metformin have been proven to suppress endothelial proliferation, migration, and angiogenesis in vitro,^10^ which is implicated in its anti‐angiogenic activity.^11^ According to results of pharmacokinetic studies, this concentration is at least 50‐fold excess over the plasma concentration of patients or experimental animals.^12^, ^13^ Thus, these results based on high concentrations may be not enough to explain the beneficial actions of metformin in cancers.

Recently, metformin's antitumour and anti‐angiogenic activities have been demonstrated to be associated with tumour‐associated macrophages (TAMs),^14^ a major component in microenvironment.^15^ TAMs in tumours always differentiate into anti‐inflammatory M2‐like phenotype in hypoxic microenvironment,^16^, ^17^ thus secreting cytokines to promote tumour progression and angiogenesis.^17^, ^18^ However, how metformin affects the differentiation of TAMs into distinct phenotypes and the underlying mechanism remain poorly understood. Given that skewing of TAM polarization is able to inhibit tumour progression,^19^ we investigated the mechanism mediating the antitumour and anti‐angiogenic effects of metformin, with focus on its previously documented immune modulatory effects.

## 2 MATERIALS AND METHODS

### 2.1 Cells and reagents

4T1 (murine breast cancer), CT‐26 (murine colorectal cancer), Renca (murine renal cancer) and human umbilical endothelial cell (HUVEC) lines were obtained from American Type Culture Collection (ATCC). Murine macrophage cell line RAW264.7 was kindly provided by the Key Laboratory of Cardiovascular Disease of Shaanxi Province. Before performing cellular experiments, all cells were confirmed to be mycoplasma free (using Mycoplasma Test Kit). Cells were cultured in the Dulbecco's Modified Eagle Medium (DMEM) supplemented with 10% foetal bovine serum (FBS) in an atmosphere of 5% CO^2^‐95% air.

Clodronate (CLO) and control liposomes were purchased from LIPOSOMA (Amsterdam, Netherlands). Recombinant interleukin (IL)‐13 was obtained from R&D SYSTEMs (Minnesota, USA). Propidium iodide (PI) was purchased from Sigma‐Aldrich (Missouri, USA).

### 2.2 Flow cytometry

2 × 10^5^ cells were seeded into 6‐well plates and cultured with increasing doses of metformin (0.1‐1 mmol/L) for 24 hours. For cell cycle analysis, cultured cells were digested with trypsin, washed by PBS and fixed in pre‐coated 70% alcohol at 4°C for 6 hours. Then, cells were incubated with RNase solution (1 mg/mL) at 37°C for 1 hour and 100 μg/mL PI for 30 minutes at room temperature (keep in dark place). For analysis of cell apoptosis, collected cells were washed and centrifuged at 2000 *g* for 5 minutes to remove cell debris. After cell counting, 5 × 10^5^ HUVECs were resuspended in pre‐coated PBS and incubated with 10 μL FITC‐conjugated annexin‐V and PI (100 μg/mL) for 30 minutes in a dark room. At least 1.0 × 10^4^ cells were analysed using the BD FACS‐Calibur cytometer (San Jose, CA) for each sample.

### 2.3 Macrophage‐conditioned medium

Briefly, 1 × 10^6^ RAW 264.7 macrophages were seeded into 6‐well plates and pre‐treated with metformin (1 mmol/L), IL‐13 (15 ng/mL) or the combination treatment. Forty‐eight hours later, supernatants were collected, centrifuged to remove cell debris and stored in the fridge (−80°C). Subsequently, macrophages were collected with a cell scraper, and the total cell number was counted for each well. After that, loading of macrophage‐conditioned medium was appropriately corrected according to the difference of macrophage number between wells (Because cell number would affect the total amount of cytokines secreted into the supernatant).

### 2.4 Cellular viability and migration assay

For assessing cellular viability, cells were seeded into 24‐well plates and subsequently cultured with metformin or conditioned medium of macrophages for 48 hours. After that, cellular viability was assessed using CCK‐8 (Cell counting kit‐8) assay kit (Dojindo, Japan) as previously described.^20^

For observation of endothelial migration in vitro, 2 × 10^6^ HUVECs were seeded and cultured in 6‐well plates until confluence. After that, well plates were vertically scratched using the tip of a 200‐μL pipette. After gently washing, 3 mL of prepared macrophage‐conditioned medium was added into duplicate per variant. Twenty‐four hours later, scratched crosses were photographed using a microscope at 100× magnification.

### 2.5 Coculture vascular sprouting assay

To observe the interaction between macrophage and ECs, a transwell insert system (with 0.4 μm pore) was employed. The pore in this size would prevent the passage of cells from one chamber to another. Initially, RAW264.7 cells were pre‐treated with IL‐13 to induce M2 polarization or in combination with 1 mmol/L metformin for 48 hours. After that, 4 × 10^4^ M2‐polarized macrophages and 2 × 10^4^ HUVECs were seeded into the upper and the lower chambers, respectively. Forty‐eight hours later, HUVECs on the lower chamber were washed, fixed and stained with haematoxylin and eosin (H&E). Finally, number of endothelial sprouts was counted under a light microscope and statistically analysed.

### 2.6 Tube formation assay

In vitro endothelial tube formation assay was performed as described.^20^ Briefly, wells of a 96‐well plate were pre‐coated with 10 μL Matrigel (at 37°C for 30 minutes for polymerization). Then, 2 × 10^4^ HUVECs were seeded into these wells, and capillary‐like network was recorded and analysed 24 hours later.

### 2.7 Animals, tumour models, and macrophage depletion

All experimental procedures were in accordance with a protocol approved by Institutional Animal Care and Use Committee of Xi'an Jiaotong University. Six‐ to eight‐week‐old BALB/c mice (about 18 g) were purchased from Experimental Animal Center of Xi'an Jiaotong University and housed in a controlled environment. 1 × 10^6^ cancer cells were subcutaneously injected to the flanks of mice. Seven days after inoculation, tumour‐bearing mice were randomly divided into different groups and then administrated with metformin (orally, 50, 100, 200, 300 mg/kg day) or in combination with CLO liposome. Maximum tumour diameters were measured every 3 days using a caliper. Three weeks after inoculation, mice were killed by injecting excessive amount of pentobarbital solution into abdominal cavity. Then, tissues of rectum and tumour were extracted.

For deletion of macrophages in vivo, tumour‐bearing mice were intraperitoneally injected with 100 mg/kg of the clodronate liposome.^19^ After that, 50 mg/kg of clodronate liposome was injected every fourth day to prevent repopulation of macrophages. CD68^+^ macrophages in both tumours and murine rectums were detected by IHC staining and counted to assess the efficiency of macrophage depletion.

### 2.8 Immunohistochemistry (IHC) and immunofluorescence (IF)

Mouse tissues were extracted, fixed, sectioned and immunostained as previously described.^20^ Briefly, for IF staining, 4% paraformaldehyde (PFA)‐fixed tissues were cut into 6 μm (for 2D confocal microscopy) or 40‐50 μm (for 3D confocal microscopy). After blocking with BSA (bovine serum albumin, 5%), sections were permeabilized with 0.2% Triton X‐100 (15 minutes), washed and stained with anti‐CD31 (Abcam), anti‐CD68, anti‐Arg‐1 and anti‐iNOS antibodies at 4°C for 24 hours. Then, sections were incubated with the corresponding secondary antibodies. For IHC staining, paraffin‐embedded mouse tissues were cut into 4 μm sections. After deparaffination and rehydration, sections were sequentially incubated with 3% H_2_O_2_, 5% BSA (blocking) and anti‐VEGF or anti‐CD68 antibodies. Subsequently, sections were incubated with the appropriate secondary antibody conjugated by horseradish peroxidase‐streptavidin (Boster, China) and counterstained with haematoxylin. The image of the whole slide was recorded using a slide scanner (Leica). For quantification, 5‐10 fields were randomly chosen and analysed. IHC score was calculated by adding intensity score to positive area score. CD31^+^, CD68^+^, CD68^+^/Arg‐1^+^ and CD68^+^/iNOS^+^ areas were measured using ImageJ software. Vascular branches, PARP^+^ apoptotic cells and CD68^+^ TAMs were directly counted and analysed. More details about antibodies can be found in the Table S1.

### 2.9 Real‐time qRT‐PCR

To detect the change of mRNA levels of VEGF, FGF‐2 and PlGF, a standardized real‐time quantitative PCR was performed according to the manufacturer's instructions (Takara). Sequences of primers used for PCR can be found in the Table Table S1.

### 2.10 Statistical analysis

All measurement data were represented as mean ± SEM (if normally distributed) or median with interquartile range (if not normally distributed). For two‐group comparison, student *t* test was used; for multiple‐group (>2) comparison, Statistical significance of the difference was defined using one‐way ANOVA (Prism5, San Diego, CA). *P *<* *.05 is considered significant; *P *>* *.05 indicates not significant.

## 3 RESULTS

### 3.1 Systematic metformin administration reduces tumour growth

To evaluate the effects of metformin on tumour progression, we implanted 4T1 cells (murine breast cancer), CT‐26 cells (murine colorectal cancer) and Renca cells (murine renal carcinoma) in BALB/c mice. When mean tumour maximum diameter reached 2‐3 mm, mice were orally administrated with metformin at doses of 50, 100, 200 and 300 mg/kg day. Administrated routine doses of metformin range from 500 to 2000 mg every day. According to the formula for interspecies dose conversion (based on body surface),^21^ this clinically‐applicated dose range equals to 65‐258 mg/kg day for mouse administration. To study the effect metformin on primary tumour growth and angiogenesis, we thus set the doses ranging from 50 to 300 mg/kg day and referred to 50 and 100 mg/kg day as the low doses and to 200 and 300 mg/kg day as the high doses.

**Figure 1:**
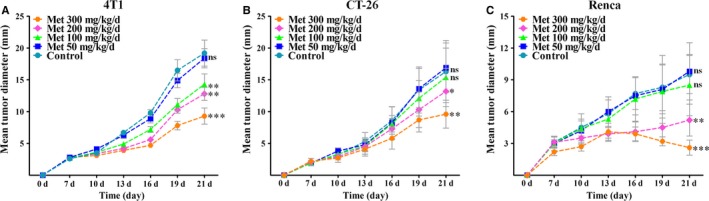
Systematic metformin administration inhibited tumour growth. A‐C, 4T1 (breast cancer, BALB/c), CT‐26 (colorectal cancer, BALB/c) and Renca (renal carcinoma, BALB/c) cells were subcutaneously injected into the flanks of mice (n = 8). Tumour‐bearing mice were orally administrated with metformin that was added to the drinking water. Maximum diameter of each tumour was measured every 3 days. Measurement data are represented as mean ± SEM (n = 8). *P <* *.05; **P <* *.01; ***P <* *.001; “ns” indicates no statistically significant difference (*P *>* *.05)

50 mg/kg day metformin exhibited no effect on the growth of all 3 tumours (Figure 1A‐C), while 100 mg/kg day metformin only resulted in a slight (but significant) reduction in 4T1 tumour growth (Figure 1A). Both high doses of metformin significantly reduced the growth of 3 tumours (Figure 1A‐C), as evidenced by the reduced maximum tumour diameter. We also found that 300 mg/kg day induced a more significant reduction in tumour growth reduction than 200 mg/kg day (Figure 1A‐C), indicating that antitumour action of metformin is dose‐dependent. In addition, 300 mg/kg day metformin significantly caused a slight regression of Renca tumour, but not 4T1 and CT‐26 tumours. Because tumour growth depends on nutrients supplied by the newly formed vessels,^22^ we explored whether metformin had inhibitory effects on tumour angiogenesis.

### 3.2 Metformin inhibits tumour angiogenesis and normalized the morphology of vasculature

**Figure 2:**
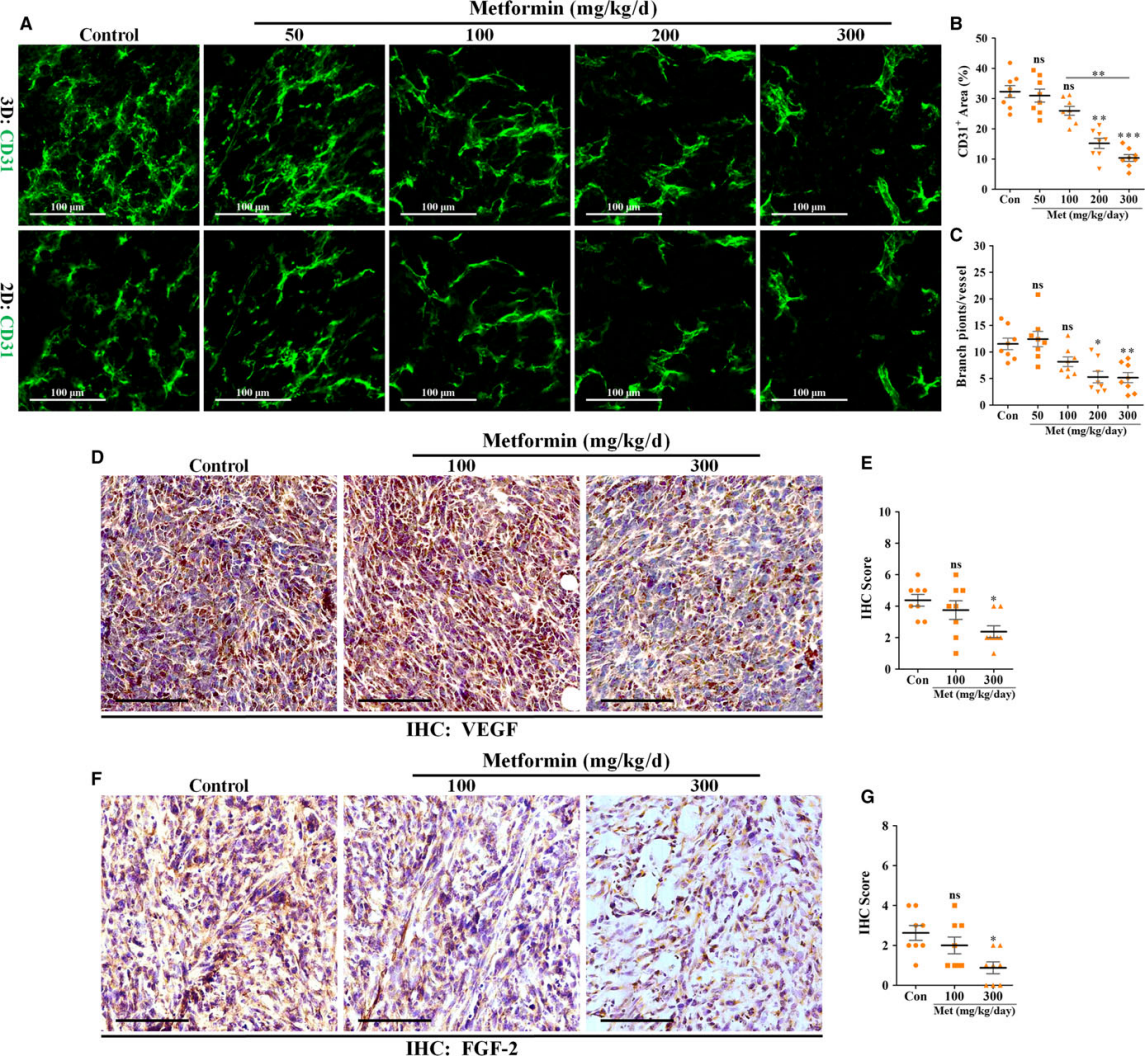
Tumour neovascularization was negatively affected by metformin in vivo. 4T1 tumours were wholly extracted 21 days after inoculation and prepared for further IF and IHC staining. A, Immunofluorescent 2D and 3D reconstruction images of CD31^+^ vessels in sections of 4T1 tumours from mice untreated or treated with metformin (mg/kg day). Scale bar: 100 μm. Quantification of B, CD31^+^ areas and C, vascular branch points of vessels in 4T1 tumours (n = 8). D and F, IHC staining for (D) VEGF and (F) FGF‐2 in sections of 4T1 tumours from mice untreated or treated with 100 or 300 mg/kg day metformin. Scale bar: 100 μm. E and G, Quantification of IHC scores (addition of intensity score and positive area score) of (E) VEGF and (G) FGF‐2 (n = 8). Measurement data are represented as mean ± SEM. *P <* *.05; **P <* *.01; ***P <* *.001; “ns” indicates no statistically significant difference (*P *>* *.05)

Cancers are characterized by a morphologically abnormal vasculature that is closely associated with the excessive angiogenesis.^23^, ^24^ 4T1 tumour sections were immunostained for CD31, an endothelial‐specific marker. Consistently, vascular architecture in metastatic breast tumour was enlarged, chaotic, disordered and with excessive vascular sprouting (Figure 2A). Treatment with low doses of metformin for 14 days did not result in a significant inhibition of 4T1 tumour angiogenesis, as evidenced by the unaffected percentage of CD31^+^ area and number of vascular sprouts per vessel (Figure 2A‐C). Conversely, high doses of metformin significantly suppressed tumour angiogenesis via a dose‐dependent manner, which was indicated by a higher significance of 300 mg/kg day than 200 mg/kg day. Furthermore, vascular architecture in 4T1 tumour was normalized to a more regular and ordered phenotype by high doses of metformin (Figure 2A).

### 3.3 Metformin‐induced anti‐angiogenesis is contributed by reduced VEGF expression

Because of the critical role of VEGF in tumour angiogenesis, we next measured the VEGF expression level in tumours by immunohistochemistry. VEGF was found to be highly expressed in tumours from untreated mice (control; Figure 2D), which at least partially explains the excessive angiogenesis in 4T1 tumours. Metformin at a dose of 300 mg/kg day, but not 100 mg/kg day, significantly reduced expression levels of both VEGF and FGF‐2 in tumours (Figure 2D‐G), indicating the involvement of VEGF and FGF‐2 signalling in metformin‐induced anti‐angiogenesis.

### 3.4 Metformin causes only a slight inhibition of EC functions

Previously published articles had demonstrated the direct inhibitory effects of metformin on EC functions.^10^, ^25^ As direct targeting is capable of inhibiting tumour angiogenesis, we thus explored if metformin mediates anti‐angiogenesis via an EC‐dependent mechanism. Under consideration of the higher accumulation of metformin in tissues than plasma,^12^ we set concentrations ranging from 0.1 μmol/L to 1 mmol/L for further in vitro experiments,^14^ which are more related to the clinically‐applicated concentration range.

**Figure 3:**
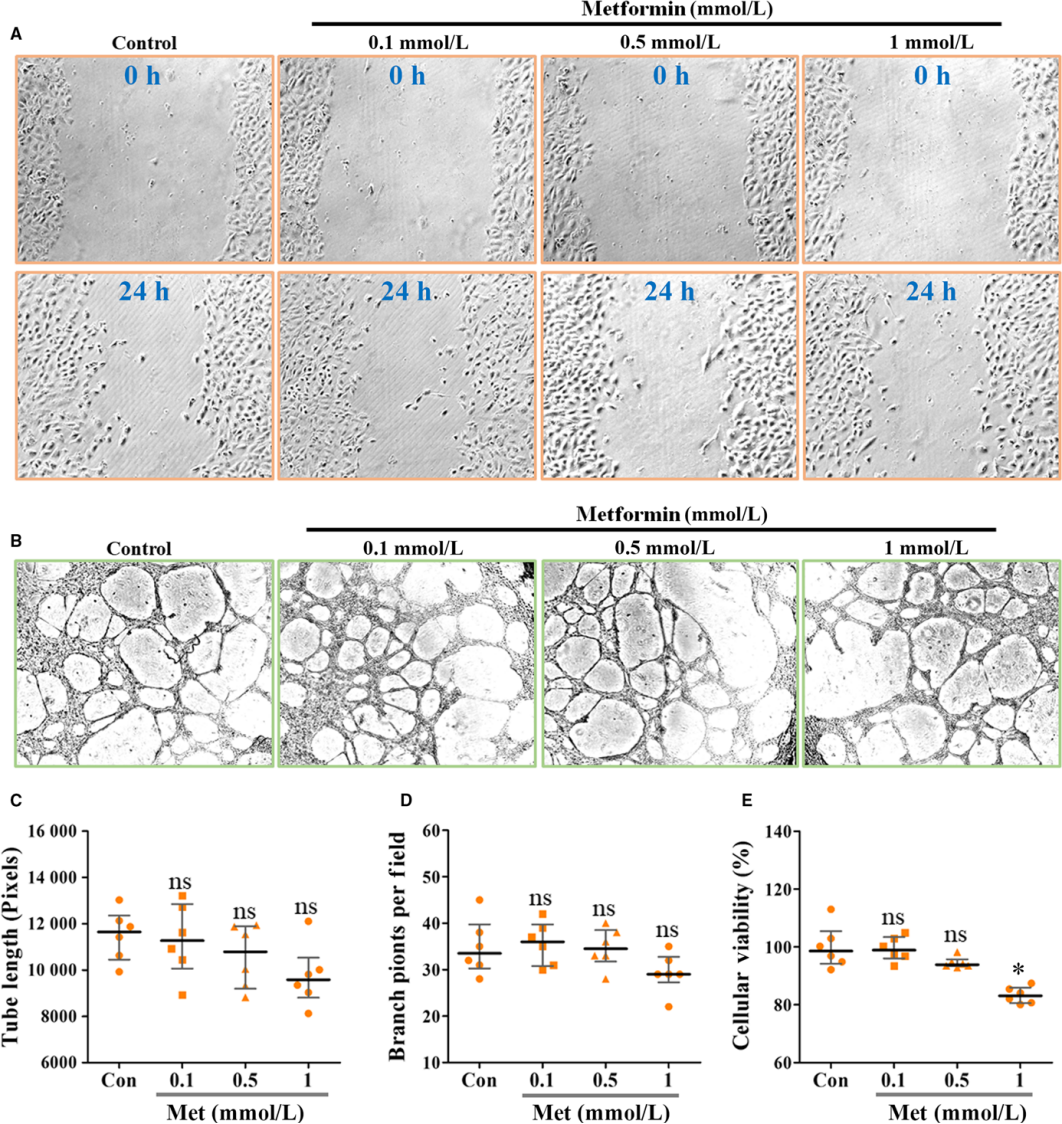
Metformin exhibited a slight inhibitory effect on EC proliferation, but not migration and tube formation. HUVECs were treated with metformin at concentrations ranging from 0.1 to 1 mmol/L. A, Representative images showing wound‐healing migration of HUVECs untreated or treated with metformin for 24 h. 100×. B, Representative images for showing HUVECs‐mediated in vitro Matrigel tube formation. Magnification: 200×. Quantifications of C, tube length and D, branch points of the formed vascular tube on Matrigel (n = 6). E, Percentage of HUVEC viability when incubated with PBS (Con) or metformin (Met; n = 6). Measurement data are represented as median with interquartile range; *P <* *.05; “ns” indicates no statistically significant difference (*P *>* *.05)

In vitro EC wound‐healing assay showed that EC migration was not apparently affected by metformin even at 1 mmol/L concentration (Figure 3A). We next performed EC tube formation assay and investigated the direct effects of metformin on in vitro EC proliferation. As shown, low concentrations (0.1 and 0.5 mmol/L) of metformin had no obvious effects on in vitro EC tube formation (Figure 3B‐D) and proliferation (Figure 3E). With 1 mmol/L metformin for 24 hours, endothelial viability, tube length and branch points were significantly reduced by <20% (viability: −17.7%; tube length: −15.3%; branch points: −15.9%) (Figure 3B‐E).

### 3.5 Metformin‐induced anti‐angiogenesis does not depend upon an EC‐autonomous mechanism

**Figure 4:**
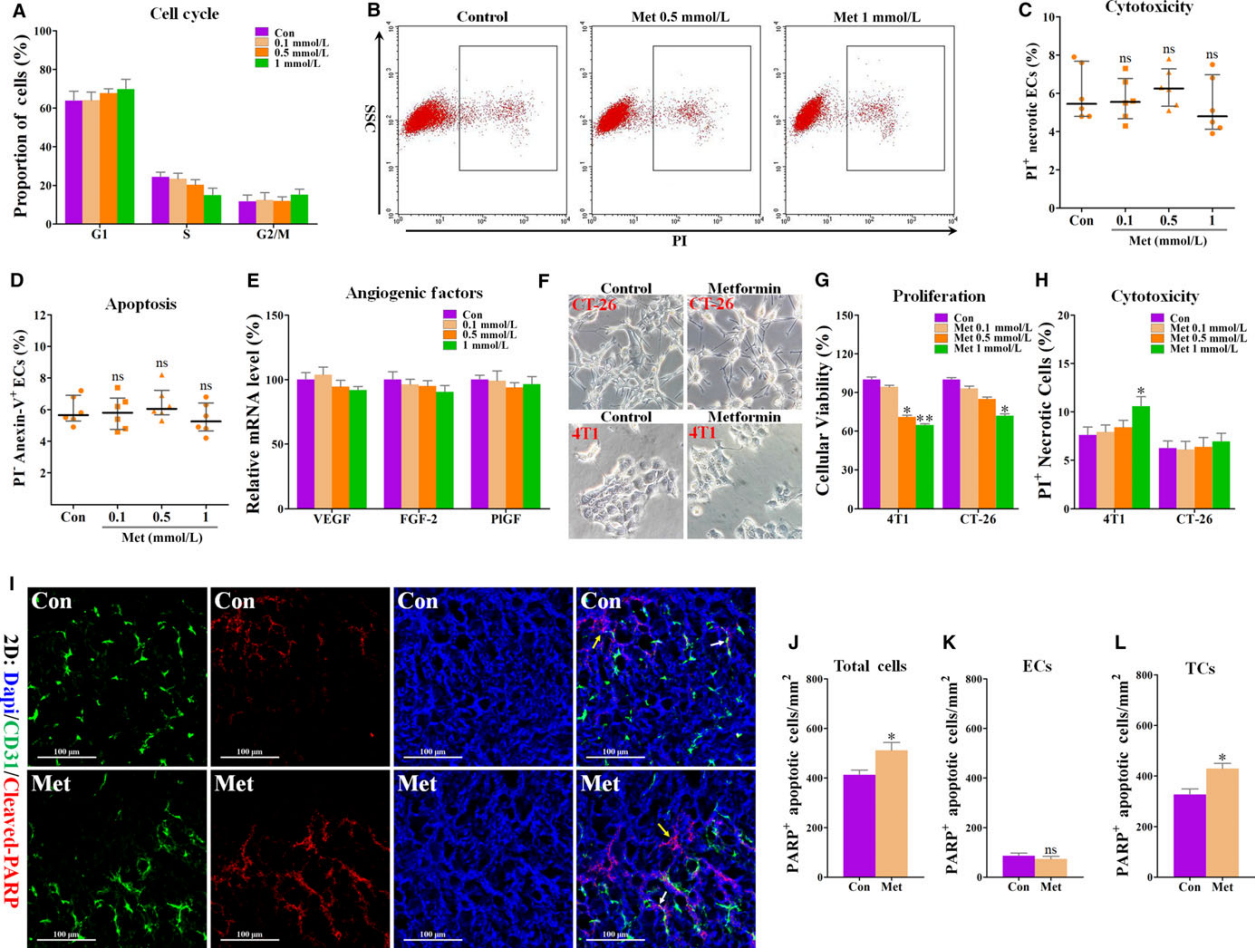
Metformin had no significant effects on cell cycle, apoptosis and angiogenic factor expressions of HUVECs. Cells were treated with PBS (Con) or metformin (Met) for 24 h and then tested for apoptosis, cell cycle, mRNA expressions of pro‐angiogenic factors and cytotoxicity of metformin. A, Proportion of ECs in different phases of cell cycle (n = 6). B, Flow cytometric analysis of PI ^+^ HUVECs treated with PBS (Con) or metformin (Met), and C, Quantification of percentage of PI ^+^ ECs for detecting cytotoxicity of metformin to ECs (% total cells; n = 6). D, Flow cytometric analysis of HUVECs stained with PI and annexin‐V, and quantification of proportion of PI ^−^/Annexin‐5^+^ ECs (n = 6). E, Real‐time PCR analysis for detecting the mRNA levels of VEGF, FGF‐2 and PlGF in HUVECs treated with metformin (n = 6). F, Representative images for showing the cellular morphology of 4T1 and CT‐26 cells treated with PBS (Control) or metformin (1 mmol/L) for 24 h. Magnification: 200×. G, Cellular viability of 4T1 and CT‐26 tumour cells (relative to control) when incubated with PBS (Control) or 1 mmol/L metformin for 24 h (n = 6). H, Percentage of PI ^+^ tumour cells (% total cells) when incubated with 1 mmol/L metformin for 24 h (n = 6). I‐L, Double staining for CD31 (green) and cleaved PARP (red), revealing increased cleaved PARP ^+^/CD31^−^ apoptotic tumour cells in metformin‐treated 4T1 tumours. White arrows indicate cleaved PARP ^+^ apoptotic ECs; yellow arrows indicate cleaved PARP ^+^ apoptotic tumour cells (TCs). Scale bar: 100 μm. Nucleus was counterstained with DAPI solution. Scale bar: 100 μm. Densities of cleaved PARP ^+^ (J) cells (K) ECs and (L) TCs (number of cells/mm^2^; n = 8). Measurement data are represented as median with interquartile range (not normally distributed data) or mean ± SEM (normal distribution data). *P <* *.05; **P <* *.01; ***P <* *.001; “ns” indicates no statistically significant difference (*P *>* *.05)

We next examined the effects of metformin on endothelial cell cycle, apoptosis and cytotoxicity. Flow cytometric analysis showed that although 1 mmol/L metformin (but not 0.1 and 0.5 mmol/L) could arrest endothelial cell cycle (Figure 4A), this effect was slight and not significant. Further annexin‐V/PI double staining and PI single staining showed similar results that 0.1‐1 mmol/L metformin did not directly affect endothelial apoptosis and showed no toxic effects on ECs (Figure 4B‐D). We also detected mRNA levels of pro‐angiogenic factors in ECs and found that metformin treatment exhibited no effects on expressions of VEGF, FGF‐2 and PlGF (Figure 4E) in HUVECs. Taken together, these data suggest that metformin‐induced anti‐angiogenesis may not depend on an EC‐autonomous manner.

### 3.6 Partial contribution of cell cytotoxicity to metformin‐mediated antitumour activity but not anti‐angiogenesis

As anti‐angiogenesis alone unlikely explained the suppressed tumour growth by metformin, we next explored if metformin had direct effects on tumour cell functions. Metformin treatment did not exhibit a significant inhibitory effect on cellular morphology of both 4T1 and CT‐26 cells (Figure 4F). We also investigated the effects of metformin on cancer cell proliferation. As shown in Figure 4G, metformin treatment suppressed in vitro proliferation of both 4T1 and CT‐26 cells, as evidenced by the reduced cellular viability. This inhibitory effect appeared to be more significant as the dose increased to 1 mmol/L, suggesting that metformin‐induced proliferative inhibition is dose‐dependent.

Inconsistent with in vivo response of tumours to metformin, only 1 mmol/L metformin significantly increased the percentage of PI^+^ 4T1 tumour cells by 2.98% (Figure 4H). However, all concentrations of metformin had no cytotoxic effect on CT‐26 tumour cells. In line with the cytotoxic effect of metformin on 4T1 cells, metformin administration exhibited only a slight (but significant) increase in density of PARP^+^ tumour cells (cleaved PARP^+^/CD31^−^) in vivo (Figure 4I‐L). Critically, metformin did not alter endothelial apoptosis (cleaved PARP^+^/CD31^+^) in 4T1 tumours (Figure 4I‐L), which is consistent with our in vitro results (Figure 4C,D). Thus, the discrepancy between metformin's antitumour and anti‐angiogenic effects implies the existence of a cell non‐autonomous mechanism in vivo.

### 3.7 Metformin skews TAM polarization from M2‐ to M1‐phenotype

**Figure 5:**
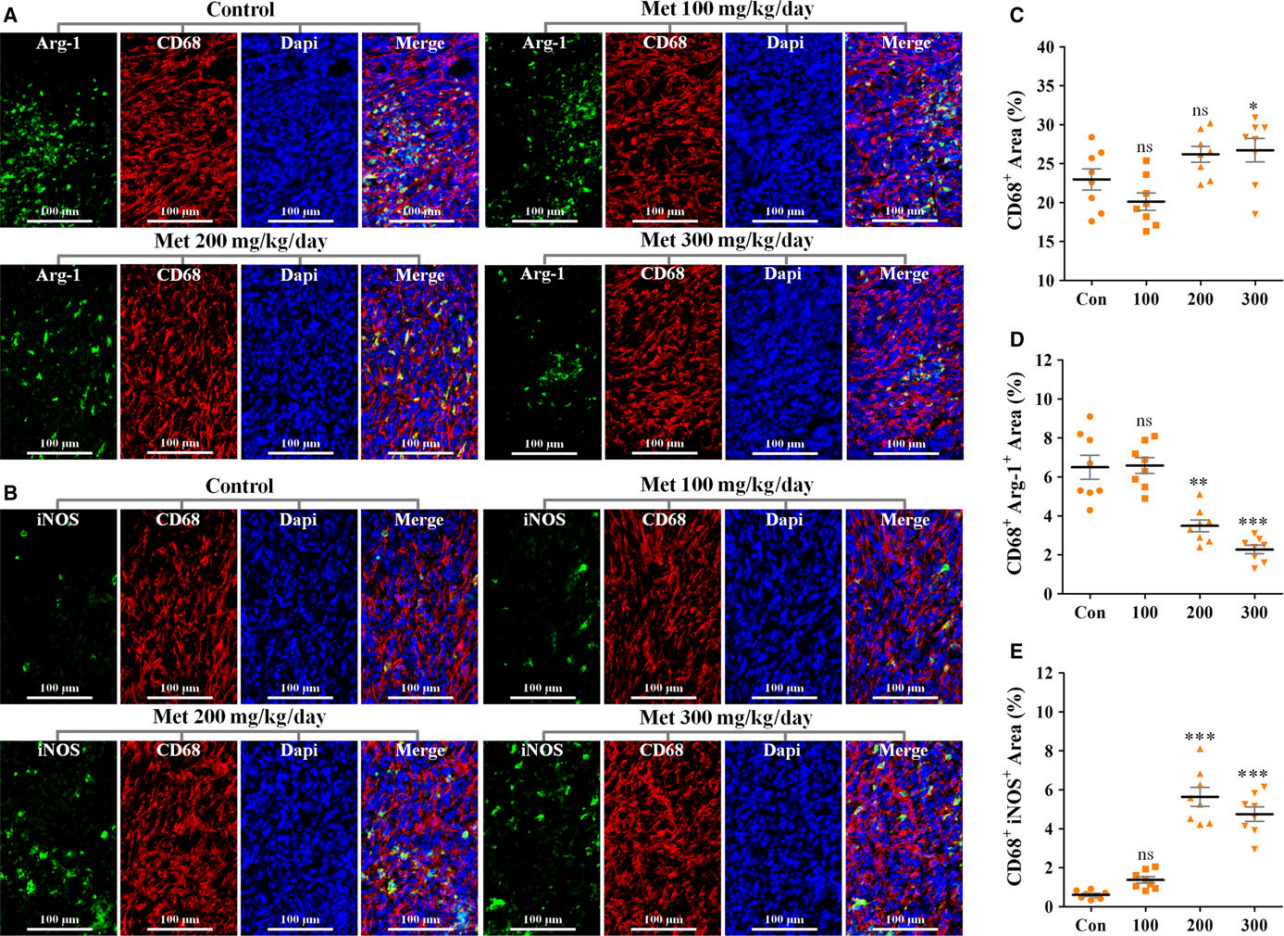
Effects of metformin on TAM polarization. A‐E, Double staining for CD68 (Red) and (A) Arg‐1 (Green) or (B) iNOS (Green), revealing decreased infiltration of CD68^+^/Arg‐1^+^ TAMs (M2‐like phenotype) and increased infiltration of CD68^+^/iNOS ^+^ TAMs (M1‐like phenotype) in 4T1 tumours from metformin‐treated mice. Nucleus was counterstained with DAPI solution. Scale bar: 100 μm. C, Quantification of CD68^+^ area (% total tumour area; n = 8). D, Quantification of CD68^+^ Arg‐1^+^ area (% CD68^+^ area; n = 8). E, Quantification of CD68^+^ iNOS ^+^ area (% CD68^+^ area; n = 8). Quantitative data are indicated as mean ± SEM. *P <* *.05; **P <* *.01; ***P <* *.001; “ns” indicates no statistically significant difference (*P *>* *.05)

We next focused on TAMs, as they greatly contribute to tumour progression and new blood vessel formation.^26^, ^27^ Consistent with previously published data,^28^ CD68^+^ TAMs congregated in large number in 4T1 tumour tissues (Figure 5A,B). However, CD68^+^ TAMs accumulation was only slightly (but significantly) increased in 4T1 tumours from 300 mg/kg day metformin‐treated mice (Figure 5C), while effects of 100 and 200 mg/kg day metformin were not significant. Because TAMs accumulation increased despite tumour growth inhibition, we next focused on the effects of metformin on TAMs polarization state. Compared to the control group, percentages of CD68^+^/Arg‐1^+^ area (M2‐phenotype) were significantly reduced in 200 and 300 mg/kg day groups but was unaffected in 100 mg/kg day group (Figure 5A,D). Conversely, accumulation of M1‐like TAMs (CD68^+^/iNOS^+^) was apparently increased in 200 and 300 mg/kg day metformin‐treated 4T1 tumours (Figure 5B,E). Overall, these data suggest that metformin skews TAMs polarization from M2‐like phenotype to M1‐like phenotype known to suppress tumour growth and angiogenesis.

### 3.8 Chemical TAM depletion abrogates the anti‐angiogenic effects of metformin

**Figure 6:**
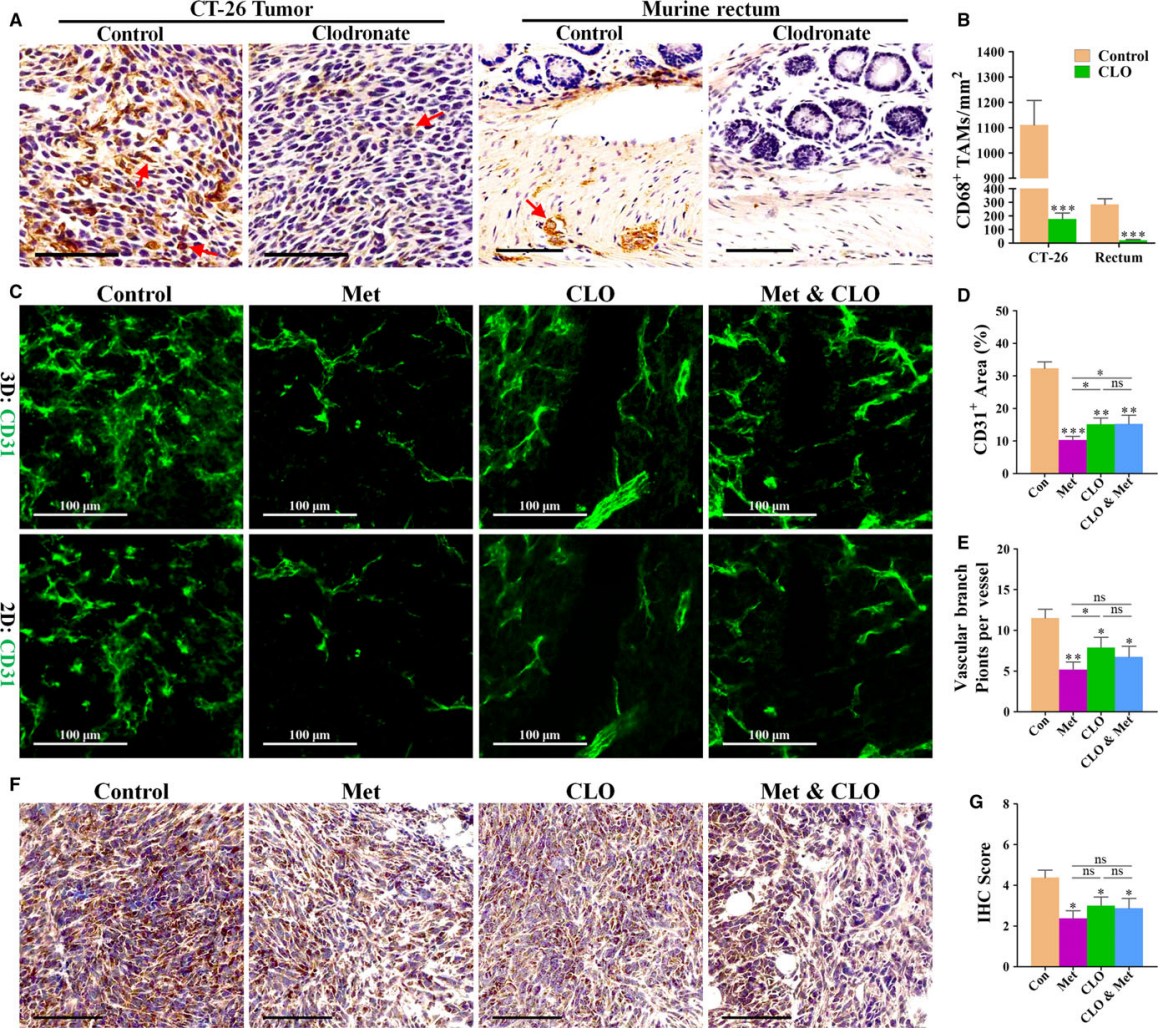
Anti‐angiogenic effect of metformin was mediated by targeting TAMs. A, Immunostaining for CD68 (brown), revealing CD68^+^ TAMs in CT‐26 tumour (left) and rectum (right) in mice, treated with vehicle (control) and clodronate liposome (clodronate). Red arrows indicate CD68^+^ TAMs distributed in tissues of 4T1 tumour and murine rectum. Scale bar: 75 μm. B, Quantification of CD68^+^ TAMs per mm^2^ in CT‐26 tumour and murine rectum (n = 8; ***P <* *.001 versus control). C, Immunofluorescent 2D and 3D reconstruction images of CD31^+^ vessels in sections of 4T1 tumours, untreated or treated with metformin (Met), clodronate (CLO) or combination treatment (Met & CLO). Scale bar: 100 μm. D and E, Quantification of (D) CD31^+^ vascular areas and (E) vascular sprouting points in 4T1 tumours from BALB/c mice (n = 8). F and G, Immunostaining for VEGF, revealing similar reduction in VEGF in metformin‐treated 4T1 tumours from mice receiving CLO or not. G, Quantification of VEGF IHC score (addition of intensity score and positive signal area) of 4T1 tumours (n = 8). Quantitative data are indicated as mean ± SD *P <* *.05; **P <* *.01; ***P <* *.001 versus control vehicle. “ns” indicates no statistically significant difference (*P *>* *.05)

To investigate if metformin‐induced anti‐angiogenesis is contributed by skewing TAM polarization, we chemically depleted macrophages in tumour‐bearing mice using CLO liposomes. To assess the depletion efficacy, tissue sections were immunostained with a CD68 antibody. As shown in Figure 6A,B, CD68^+^ macrophages were reduced by 84% and 92% in CT‐26 tumours and murine rectum from metformin‐treated mice (Figure 6A,B), respectively, indicating the successful depletion of macrophage by CLO. Further characterization of CD31^+^ tumour vessels showed (Figure 6C,E) that TAM depletion decreased CD31^+^ area (% total area) and vascular branch points (per vessel), indicating that TAMs were predominantly of the angiogenesis‐promoting phenotype (M2‐like) in tumours. Chemical depletion of TAMs by CLO significantly increased CD31^+^ vascular area in tumours from metformin‐treated mice (Figure 6D), suggesting that metformin also abrogated M1‐like TAMs‐induced anti‐angiogenesis. Not unexpectedly, reduction level of VEGF was comparable between tumours from single metformin‐treated and combined metformin/CLO‐treated mice (Figure 6F,G). Thus, the anti‐angiogenic activity of metformin may rely on its ability to skew TAMs polarization from M2‐like to M1‐like phenotype.

### 3.9 Metformin suppresses tumour proliferation and angiogenesis promoted by M2‐like macrophages

**Figure 7:**
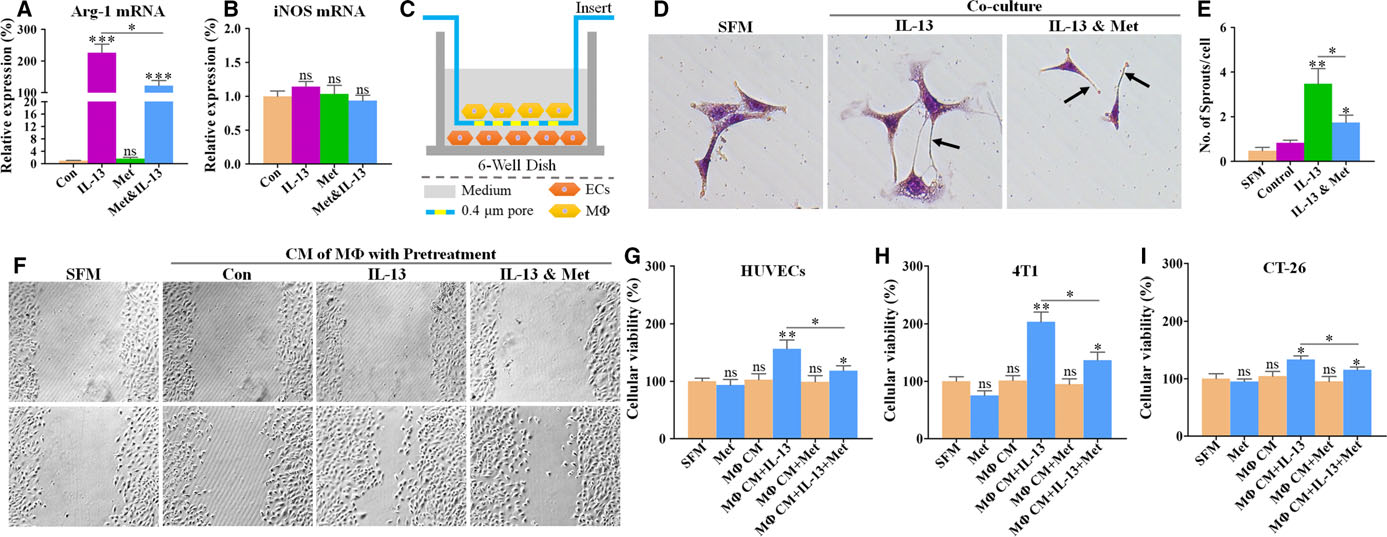
Effects of metformin on tumour proliferation and angiogenesis promoted by M2‐like macrophages. A and B, Quantitative RT‐PCR for detecting the mRNA expression levels of (A) Arg‐1 and (B) iNOS in RAW264.7 macrophages (n = 6). RAW264.7 cells were cultured with vehicle, 1 mmol/L metformin, 15 ng/mL IL‐13 or both for 72 h. C, Representative image showing a Transwell Insert System (0.4 μm), coculturing endothelial cells (ECs; on the bottom chamber) with M2‐polarized RAW264.7 macrophages (MΦ; on the lower chamber). D, Representative images showing the cellular morphology of HUVECs cocultured with macrophages (MΦ), pre‐treated with IL‐13 (15 ng/mL) or in combination with metformin (Met; 1 mmol/L). Black arrows indicate EC‐mediated vascular sprouting. Magnification: 400×. E, Quantification of vascular sprouting, revealing metformin pre‐treatment‐mediated abrogation of endothelial sprouting that was promoted by M2‐polarized macrophages (n = 6). F‐I, Representative images showing (F) endothelial wound‐healing migration and proliferations of (G) HUVECs, (H) 4T1 and (I) CT‐26 tumour cells, cultured in serum‐free medium (SFM) or conditioned medium (CM) from RAW264.7 macrophages (M). Macrophages were pre‐treated with PBS (Con), 15 ng/mL IL‐13 or in combination with 1 mmol/L metformin (Met) for 48 h (n = 6). Quantitative data are indicated as mean ± SEM. *P <* *.05; **P <* *.01; ***P <* *.001. “ns” indicates no statistically significant difference (*P *>* *.05)

We next analysed the impact of metformin on M2‐like macrophage‐induced promotion of tumour proliferation and angiogenesis in vitro. Macrophages treated with IL‐13 assume a M2‐like phenotype.^14^ When stimulated with IL‐13, RAW264.7 macrophages were differentiated into M2‐like rather than M1‐like macrophages, as evidenced by intensely increased mRNA expression of Arg‐1 (Figure 7A), a M2 marker. In the presence of IL‐13, metformin significantly decreased the expression level of Arg‐1, while having no effect on expression of iNOS, a marker for M1, indicating that metformin is potential of inhibiting cytokine‐induced M2 polarization of macrophages.

To get a deep understanding of metformin‐induced anti‐angiogenesis by targeting TAMs‐ECs interaction, macrophages were cocultured with ECs using a Transwell Insert System (Figure 7C). When cocultured with M2‐polarized macrophages, ECs generated more and longer vascular sprouts than those not cocultured (Figure 7D,E). In the absence of IL‐13, metformin pre‐treatment exhibited a significant inhibition of ECs‐mediated vascular sprouting, suggesting that metformin is potential of suppressing M2‐like macrophage‐promoted endothelial angiogenesis.

Furthermore, conditioned medium (CM) were used for determining the inhibitory effects of metformin pre‐treatment on functions of ECs and tumour cells, which were promoted by M2‐polarized macrophages. Tumour cells and ECs viabilities and endothelial migration ability were greatly increased after incubation with CM of M2‐polarized rather than unpolarized macrophages (Figure 7F‐H). These effects were further weakened when incubated with CM from metformin‐pre‐treated macrophages (Figure 7F‐H). These findings that metformin inhibited M2‐polarized macrophages‐promoted tumour proliferation and angiogenesis indicate that the antitumour and anti‐angiogenic effects of metformin relied on macrophage immunity.

## 4 DISCUSSION

Immune‐mediated antitumour effects by metformin had been demonstrated in various models of cancer,^29^ but it remains unclear how metformin exerts its effects by targeting immune cells in tumour microenvironment.^30^ In this paper, we identified an effect of metformin on TAMs polarization, which helps explain its anti‐angiogenic and antitumour actions. Using in vivo malignant cancer models, we show that anticancer and anti‐angiogenic activities of metformin are not or partially reliant on direct inhibition of cellular functions, but primarily rely on an effect on macrophage polarization.

The novelty of this paper is the identification of a metformin's effect to increase accumulation of M1‐like macrophages. We show that TAMs‐mediated anti‐angiogenic effects of metformin are mediated by dual actions of the drug. One action, metformin decreased the accumulation of M2‐TAMs in microenvironment,^14^ thus impeding M2‐like macrophage‐induced angiogenic promotion. Another action, metformin indirectly inhibited tumour angiogenesis by increasing accumulation of M1‐TAMs. The latter mechanism is validated by the fact that CLO‐induced TAMs depletion enhanced angiogenesis in tumours from metformin‐treated mice. In fact, CLO chemically eliminated M2‐like TAMs in microenvironment while simultaneously depleting M1‐like TAMs. Thus, metformin‐induced anti‐angiogenic effect was partially abrogated due to loss of M1‐TAMs‐induced anti‐angiogenesis. Although metformin slightly increased the accumulation of total TAMs, its degree was lower than the degree of metformin‐induced skewing of TAMs from M2 to M1 phenotype.The microenvironment within malignant tumours supports tumour progression and angiogenesis 31, 32 and mediates the recruitment of immune cells, such as regulatory and effector T cells and macrophages.^33^ Macrophages that infiltrate tumours, also called TAMs, are the major component of microenvironment.^16^ Most importantly, targeting TAMs by skewing the polarization state had been demonstrated to be more effective than direct inhibition of cellular functions.^34^ Based on these evidences, targeting TAMs has been widely accepted as one of the most promising therapeutic strategies for treating cancers.^15^ Consistent with Ding's results 14 basing on Lewis lung cancer model, we reported similar findings that metformin restrained cytokine‐induced M2‐polarization in vitro. Besides, we also observed metformin‐induced inhibition of M1‐TAMs accumulation in a metastatic breast cancer model other than lung cancer model, which is an expansion to Ding's findings. These data suggest that metformin may be widely available for targeting TAMs.

Recently, CD8^+^ T cell, another immune infiltrate in microenvironment, has been reported to be critical for metformin‐induced antitumour activities.^29^ This lymphocyte‐mediated effect was caused by increased infiltration of CD8^+^ T cells in tumour microenvironment. Although the number of tumour‐infiltrating CD8^+^ T cells is a critical prognostic factor for cancers,^35^, ^36^ whether these T cells can effectively inhibit tumour progression is closely related to the polarization status of TAMs. It has been well documented that M2‐like TAMs promote tumour progression by inhibiting the antitumour effects of CD8^+^ T cells.^15^ Together with our results, it could be boldly deduced that, in addition to increasing T effector cell infiltration, metformin's antitumour effect also greatly depends on the skewing of TAMs polarization. This speculation should be validated in the further investigation.

Metformin is best known as an AMPK activator,^37^ but if AMPK activation is involved in metformin‐induced effects on TAM polarization remains unclear. It was reported that AMPK signalling was closely involved in inflammatory cytokine‐mediated polarization of macrophages.^38^ Similarly, AICAR, an AMPK‐specific activator, was found to significantly inhibit cytokine‐induced M2‐polarization of macrophages in vitro.^14^ Despite these evidences, this mechanism has not yet been definitely elucidated in models of in vivo experiments. At least, attentions should be focused on in situ observations of AMPK activation status in TAMs. Besides, in the previously published articles,^39^ metformin had been shown to ameliorate hypoxic tumour microenvironment by elevating tumour blood perfusion. It is well known that hypoxia plays a crucial role in regulating polarization of TAMs in tumour microenvironment.^40^ Although this mechanism was not investigated in this report, skewing of TAMs polarization might have been due to metformin‐induced amelioration of hypoxic tumour microenvironment.

In the current paper, metformin inhibited tumour growth and angiogenesis by an indirect mechanism, which is different from the previously published articles that focused on metformin's direct effects on cellular functions.^7^, ^41^, ^42^ High concentrations (equal or higher than 2 mmol/L) of metformin always exhibited significant inhibitory effects on tumour cell migration, proliferation and invasion, whereas low concentrations (<2 mmol/L) of metformin exhibited only a slight inhibitory effect.^7^ Our in vitro results are consistent with these reported data basing on low concentrations of metformin.^29^ Plasma concentrations of metformin are estimated to be consistently <0.2 mmol/L in diabetic patients and experimental animals.^12^, ^13^, ^43^ Although tissue accumulation of metformin would result in a higher concentration in tissue than in plasma, tissue concentration of metformin was found to be <1 mmol/L.^43^ Therefore, low concentrations of metformin may be more appropriate for exploring its antitumour activities in vitro.

The findings presented here from mechanistic experiments have implications for application of metformin as a clinical treatment for cancers. As metformin is a clinically approved drug, its property to induce inhibition of tumour growth and angiogenesis will make this agent more attractive for treatment of malignant tumours. Further efforts should be made to offer pathological evidences and validate the association between ameliorated hypoxic microenvironment and skewing of macrophage polarization.

## CONFLICT OF INTERESTS

No potential conflict of interests were disclosed by the authors.
