# Supplementary material for: Metformin's antitumour and anti‐angiogenic activities are mediated by skewing macrophage polarization
Source: J Cell Mol Med. 2018 May 4;22(8):3825–36. doi: 10.1111/jcmm.13655 (PMC6050465; doi:10.1111/jcmm.13655)
Supplement: Supplementary file 1 [file JCMM-22-3825-s001.docx]

Table 1. Primers and antibodies

| **Primers** | | |
| --- | --- | --- |
| **Gene** | **Forward Primer** | **Reverse Primer** |
| GAPDH for Human | GGTGAAGGTCGGAGTCAACG | ATACTTCTCATGGTTCACACC |
| GAPDH for Mouse | GAGAGTGTTTCCTCGTCCCG | ACTGTGCCGTTGAATTTGCC |
| FGF-2 | GGCTGCTGGCTTCTAAGTGT | TCTGTCCAGGTCCCGTTTTG |
| PlGF | GTCTCACATATTCAGTCCGTCC | TTGGCTGTCTTTATCGGCAC |
| VEGF | GTGCAGGCTGCTGTAACGAT | GGGATTTCTTGCGCTTTCGT |
| **First Antibodies** | | |
| **Antibody** | **Company** | **Concentration** |
| IF: CD31 | Abcam | 1 μg/ml |
| IF: CD68 | Santa | 1.5 μg/ml |
| IF: Arg-1 | Abcam | 2 μg/ml |
| IF: iNOS | Abcam | 2 μg/ml |
| IF: Cleaved-PARP | Cell Signaling | 3 μg/ml |
| IHC: FGF-2 | ProteinTech | 1.5 μg/ml |
| IHC: VEGF | ProteinTech | 1.5 μg/ml |
| **Secondary Antibodies** | | |
| Goat anti-Rabbit Fluor 488 | Invitrogen | 2.5 μg/ml |
| Donkey anti-Mouse Fluor 546 | Invitrogen | 2.5 μg/ml |
